# JAK Inhibitor-Induced Improvement of IgA Nephropathy in a Patient With Polycythemia Vera: A Case Report

**DOI:** 10.1016/j.xkme.2026.101376

**Published:** 2026-04-27

**Authors:** Mayumi Takahashi-Kobayashi, Hidekazu Nishikii, Tatsuya Shimizu, Yoko Kaneko, Mamiko Sakata-Yanagimoto, Joichi Usui

**Affiliations:** 1 Department of Nephrology, Institute of Medicine, University of Tsukuba, Tsukuba, Ibaraki, Japan; 2 Department of Hematology, Institute of Medicine, University of Tsukuba, Tsukuba, Ibaraki, Japan; 3 Division of Cell Regulation, Center of Experimental Medicine and Systems Biology, The Institute of Medical Science, The University of Tokyo, Tokyo, Japan; 4 Department of Nephrology, Tsukuba Central Hospital, Ushiku, Ibaraki, Japan

**Keywords:** Case report, JAK inhibitors, JAKi, IgA nephropathy, IgAN, polycythemia vera, PV

## Abstract

Polycythemia vera (PV) is a myeloproliferative neoplasm characterized by the clonal expansion of hematopoietic stem cells, commonly caused by pathogenic variants in Janus kinase 2 (JAK2). Although PV primarily affects the hematologic system, various renal manifestations have been reported, including rare cases of glomerulopathies. We describe a 73-year-old woman with PV harboring a *JAK2*^V617F^ mutation who developed hematuria, proteinuria, and renal dysfunction. Kidney biopsy revealed IgA nephropathy (IgAN) with cellular crescents. Because corticosteroid therapy was initially avoided due to concern for exacerbating PV, treatment was initiated with a JAK inhibitor, ruxolitinib. Over the following year, urinary protein levels decreased and serum creatinine stabilized, suggesting that JAK inhibition may have contributed to an improvement in IgAN activity. Two years of tapering corticosteroid therapy later resulted in clinical remission. To our knowledge, this is the first reported case of IgAN showing improvement after JAK inhibitor therapy in a patient with PV. Proposed mechanisms include suppression of IL-6/IL-11–driven platelet-derived growth factor production leading to reduced mesangial proliferation, and modulation of aberrant hematopoietic IgA production. This case suggests that JAK inhibition may not only normalize hematopoiesis in PV but also modify glomerular inflammation in IgAN, particularly in patients with hematologic comorbid conditions.

## Introduction

Polycythemia vera (PV) is a chronic myeloproliferative neoplasm characterized by clonal expansion of hematopoietic stem cells, resulting in excessive production of erythrocytes and, to a lesser extent, leukocytes and platelets.^1^ The majority of patients harbor a somatic gain-of-function mutation in the Janus kinase 2 (*JAK2*) gene, most commonly *JAK2*^V617F^, which leads to constitutive activation of the Janus kinase–signal transducer and activator of transcription (JAK-STAT) signaling pathway and cytokine-independent hematopoiesis.^2^ As a stem cell-derived disorder, PV originates at the level of multipotent hematopoietic stem cells and is associated with increased risk of thrombosis, progression to myelofibrosis or acute leukemia, and organ dysfunction due to hyperviscosity.^3^ We report a patient with IgA nephropathy (IgAN) complicated by PV, in whom clinical improvement was achieved after administration of the JAK inhibitor, ruxolitinib. This case may provide novel insight into the pathophysiology of IgAN, a disease for which the mechanisms are only partially understood. JAK inhibitors are increasingly recognized as immunomodulatory agents with systemic effects, and their therapeutic applications have expanded beyond hematologic disorders to include autoimmune and inflammatory diseases involving the joints, skin, gastrointestinal tract, lungs, and kidneys.^4^ Given their role in suppressing cytokine-driven inflammation via the JAK-STAT signaling pathway, JAK inhibitors may offer therapeutic potential in glomerular diseases such as IgAN, particularly in cases complicated by coexisting clonal hematologic conditions.

## Case Report

A 73-year-old woman was referred to our hospital for evaluation of progressive leukocytosis by her primary hospital, where she had been followed for hypertension, atrial fibrillation, and previously treated hepatitis C. She was asymptomatic, with white blood cell count 16,770/μL, hemoglobin 15.5 g/dL, hematocrit 50.0%, platelet count 32.8 × 10^4^/μL, and serum erythropoietin 1.3 mIU/mL. She was thus subsequently diagnosed with PV based on bone marrow findings, with confirmation of a *JAK2*^V617F^ mutation. One year after the diagnosis of PV, urinalysis showed isolated occult blood (1+). A year later, both proteinuria (2+) and presence of occult blood (2+) had progressed, while serum creatinine remained stable at 0.85 mg/dL. There were no notable physical findings such as rash or arthralgia. Antinuclear antibody, antineutrophil cytoplasmic antibody, and anti-glomerular basement membrane antibody were negative, and complement (C3, C4) and immunoglobulin levels were within the normal range. Within 2 months, proteinuria worsened to 3+ and creatinine increased to 1.11 mg/dL, eventually peaking at 1.39 mg/dL, prompting hospitalization and kidney biopsy. The peak urinary protein-creatinine ratio was 2.7 g/g creatinine. Kidney biopsy revealed 12 glomeruli, including 1 with global sclerosis. Among the remaining glomeruli, focal mesangial proliferation was seen, with 2 having cellular crescents, 1 with a fibrous crescent, and 1 showing a segmental sclerotic lesion. In the tubulointerstitial compartment, patchy tubular atrophy and interstitial expansion were observed, accompanied by moderate interstitial inflammatory cell infiltration. In the vascular compartment, mild fibrous intimal thickening was noted in arcuate and interlobular arteries. No arteriolar hyalinosis was observed. Immunofluorescence microscopy revealed 1+ granular IgA deposition in the mesangial area and trace C3c (±), while IgG, IgM, C1q, and C4 were negative. These findings were consistent with a diagnosis of focal mesangial proliferative glomerulonephritis, IgAN with cellular crescents, Oxford classification M0S1E0T0C1. Although corticosteroid therapy was justified based on the renal biopsy and declining kidney function, persistent leukocytosis in the setting of PV raised concern that steroids might further increase leukocyte counts and complicate assessment of PV activity. There were no absolute contraindications, including infection—there was no fever or clinical focus, and laboratory evaluation revealed no evidence of sepsis—but we prioritized PV management for safety and treatment balance. A JAK inhibitor, ruxolitinib, was initiated, resulting in a reduction of proteinuria to <0.5 g/d within 1 year, with white blood cell and hemoglobin levels stabilizing at ∼9,000/μL and 11 g/dL, respectively. Although creatinine stabilized at ∼1.5 mg/dL, the persistence of active urinary abnormalities in the context of crescentic IgAN prompted initiation of oral prednisolone (30 mg/d) 1 year after biopsy. Prednisolone was tapered over 17 months, during which time the patient achieved resolution of proteinuria (<0.3 g/d), disappearance of hematuria, and a stable serum creatinine level.

## Discussion

Although PV primarily affects the hematopoietic system, a variety of renal manifestations have been reported in association with the disease. These include not only hemodynamic and thrombotic complications, such as renal infarction and venous thrombosis, but also glomerular pathologies. Case reports have described coexisting glomerulopathies, such as focal segmental glomerulosclerosis, membranoproliferative glomerulonephritis, and IgAN.^5^ A review of the literature identified approximately 18 reported cases of PV complicated by biopsy-proven IgAN, in which *JAK2* mutations were confirmed in those for which testing was performed, consistent with the diagnosis of PV (Table 1).^5^, ^6^, ^7^, ^8^, ^9^, ^10^, ^11^, ^12^, ^13^, ^14^ However, to our knowledge, none of these prior PV cases were treated with a JAK inhibitor, making our case the first to demonstrate clinical improvement of IgAN after JAK inhibition. A single case of psoriatic arthritis-associated IgAN responding to tofacitinib has been reported,^15^ but not in the setting of PV.

**Table 1:** Previously Reported Cases of IgA Nephropathy Associated With Polycythemia Vera

Reference	Age at Biopsy (y)	Sex	*JAK2* V617F Mutation	Time From Diagnosis of PV to Diagnosis of IgAN (mo)	cr (mg/dL)	UP (g/d)	Treatment	Duration of Follow-Up (mo)	Outcome cr (mg/dL)	Outcome UP (g/d)
Kim^6^ (1994)	56	Male	NA	NA	1.3	7.8	NA	NA	NA	NA
Kasuno^7^ (1997)	35	Male	NA	60	1.6	4	ACG, PSL, RP	NA	1.2	1.5
Kasuno^7^ (1997)	51	Male	NA	Simultaneously	1	2.8	BUS, RP	NA	NA	NA
Kwon^8^ (1999)	32	Male	NA	NA	10	5.3	NA	NA	HD	HD
Chung^9^ (2002)	46	Male	NA	Simultaneously	2.7	9.14	ACEi, CCB, HU	12	Not improved	2.7
Yaguchi^10^ (2005)	55	Male	NA	204	2.5	6.9	PSL	38 d	NA	3.4
Tian^11^ (2011)	55	Male	NA	NA	1.15	13.43	ARB, CCB, MMF, RP	NA	0.9	1.5
Chen^12^ (2015)	57	Male	NA	72	1.22	2.8	ACG, ARB, CCB, HU	NA	NA	NA
Yang^5^ (2022)	19	Male	−	12	0.71	0.52	ARB, HU	60	0.86	0.31
Yang^5^ (2022)	55	Female	+	6	0.81	1.6	ARB, HU	101	1.01	0.3
Wang^13^ (2023)	22	Male	−	Simultaneously	1.09	11.76	ARB, HU, PSL	31	1.02	0.12
Wang^13^ (2023)	40	Male	−	−2	1.05	9.63	ARB, HU, TW, PSL	30	1.17	0.81
Wang^13^ (2023)	55	Male	+	11	1.91	2.1	ACEi, HU, TW	48	8.19	1.8
Wang^13^ (2023)	33	Male	+	Simultaneously	0.75	1.25	ARB, HU, interferon	14	0.75	0.52
Wang^13^ (2023)	70	Male	NA	275	1.19	2.06	ACEi, HU, TW	82	3.53	1.66
Wang^13^ (2023)	73	Male	−	36	1.28	0.73	ARB, interferon	48	1.14	0.81
Wang^13^ (2023)	51	Male	NA	−168	0.95	0.25	HU, TW	144	1.35	0.29
Rajasekar^14^ (2024)	66	Male	+	Simultaneously	2.6	3.83	ARB, HU	2	2.5	1.74
Present case (2025)	75	Female	+	12	1.39	2.7	ARB, HU→JAKi, PSL	48	1.5	<0.15

ACG, anticoagulant; ACEi, angiotensin-converting enzyme inhibitor; ARB, angiotensin receptor blocker; BUS, busulfan; CCB, calcium channel blocker; cr, creatinine; HU, hydroxyurea; JAKi, Janus kinase inhibitor; MMF, mycophenolate mofetil; NA, not available; PSL, prednisolone; PV, polycythemia vera; RP, regular phlebotomies; TW, *Tripterygium wilfordii*; UP, urinary protein.

The novelty of this case lies in the observation that ruxolitinib treatment for PV was associated with reduced proteinuria and stabilization of kidney function before corticosteroid initiation. Although corticosteroids were considered appropriate based on renal biopsy findings and declining kidney function, concerns about exacerbating PV-related leukocytosis led us to prioritize PV-directed therapy. Ruxolitinib unexpectedly resulted in marked improvement in proteinuria and hematologic stability, and steroids were introduced later for persistent urinary abnormalities. The early improvement prior to steroid initiation suggests a potential disease-modifying effect of JAK inhibition.

The pathophysiology of PV and IgAN differs fundamentally—PV is a clonal hematologic neoplasm driven by *JAK2* mutations, whereas IgAN is a glomerular disease mediated by mucosal immune dysregulation and immune complex deposition. However, both diseases may share elements of dysregulated cytokine signaling and hematopoietic immune cell dysfunction. The convergence of these pathways may underlie the unique response observed in this case. Emerging evidence has also suggested that the JAK-STAT signaling pathway may be involved in the pathogenesis of IgAN itself.^16^ In particular, interleukin-6 (IL-6), which signals through JAK1 and JAK2, has been shown to promote mesangial proliferation and enhance the production of galactose-deficient IgA1.^17^^,^^18^ Elevated expression of phosphorylated STAT3 has been reported in kidney tissue from IgAN patients,^19^^,^^20^ supporting the hypothesis that JAK-STAT activation may contribute to disease progression. These insights provide a mechanistic rationale for exploring JAK inhibitors as a potential therapeutic strategy in IgAN.

Recent experimental evidence also supports the possibility that JAK inhibition may exert direct renoprotective effects at the podocyte and glomerular basement membrane level. Studies in models of diabetic kidney disease have shown that inhibition of JAK/STAT signaling by ruxolitinib reduces albuminuria, decreases inflammatory mediators such as TGF-β1, NF-κB, and TNF-α, and improves podocyte structural integrity by restoring autophagy and reducing apoptosis.^21^ These data provide biological plausibility for the observed reduction in proteinuria in our patient after JAK inhibition. We propose 3 potential mechanisms through which JAK inhibition may have improved IgAN in this case. First, inhibition of the JAK-STAT signaling pathway may have led to decreased production of platelet-derived growth factor, a key mediator of mesangial cell proliferation and extracellular matrix deposition in mesangial proliferative glomerulonephritis. IL-6 and IL-11, which are upstream activators of platelet-derived growth factor expression, signal through JAK-STAT pathways; thus, blocking this axis may have attenuated glomerular inflammation and mesangial expansion. Second, recent experimental studies using IgA1-producing cell lines derived from the tonsils of patients with IgAN have demonstrated that leukemia inhibitory factor stimulates overproduction of galactose-deficient IgA1 through aberrant activation of the JAK2-STAT1 pathway and that JAK2 inhibitors can effectively suppress this pathogenic glycosylation process.^22^ This finding provides a mechanistic basis for the possibility that JAK inhibition could directly target IgAN pathogenesis. Third, given the hematopoietic origin of IgA production—supported by studies demonstrating resolution of IgAN after bone marrow transplantation and reduced recurrence after transplantation of kidneys from donors without IgAN—JAK inhibition may have acted directly on abnormal hematopoietic progenitors, thereby reducing the production of pathogenic IgA1. This would represent a disease-modifying effect, beyond simple immunosuppression or control of PV.

In this patient, ruxolitinib therapy proved effective, underscoring the potential role of JAK inhibition not only in the management of PV but also in the modulation of glomerular disease activity in IgAN. However, ruxolitinib cannot be recommended as a substitute for corticosteroids, which remain the standard therapy for high-risk IgAN. Further studies are required to clarify whether JAK inhibition has a therapeutic role in IgAN.

Several limitations must be acknowledged. The lack of serial renal biopsy precludes direct histologic confirmation of response, and the subsequent introduction of corticosteroid therapy confounds the attribution of clinical improvement solely to JAK inhibition. Additionally, no immunologic or molecular biomarkers were available to assess the downstream effects of JAK inhibition on cytokine profiles or IgA production. Nonetheless, this case provides a novel perspective on the interplay between myeloproliferative neoplasms and immune-mediated glomerular disease and supports further investigation of JAK-STAT–targeted therapies in IgAN, particularly in patients with coexisting hematologic or hyperinflammatory conditions.
